# Plasma p-tau181, neurofilament light chain and association with cognition in Parkinson’s disease

**DOI:** 10.1038/s41531-022-00384-x

**Published:** 2022-11-12

**Authors:** Lucia Batzu, Silvia Rota, Abdul Hye, Amanda Heslegrave, Dhaval Trivedi, Lucy L. Gibson, Chloe Farrell, Pavlos Zinzalias, Alexandra Rizos, Henrik Zetterberg, K. Ray Chaudhuri, Dag Aarsland

**Affiliations:** 1 Department of Basic and Clinical Neurosciences, Institute of Psychiatry, Psychology and Neuroscience, King’s College London, London, UK; 2 Parkinson’s Foundation Centre of Excellence, King’s College Hospital, London, UK; 3 Department of Neuroimaging, Institute of Psychiatry, Psychology and Neuroscience, King’s College London, London, UK; 4 Department of Old Age Psychiatry, Institute of Psychiatry, Psychology and Neuroscience, King’s College London, London, UK; 5 Department of Neurodegenerative Disease, UCL Institute of Neurology, Queen Square, London, UK; 6 UK Dementia Research Institute at UCL, London, UK; 7 Clinical Neurochemistry Laboratory, Sahlgrenska University Hospital, Mölndal, Sweden; 8 Department of Psychiatry and Neurochemistry, Institute of Neuroscience and Physiology, the Salhgrenska Academy at the University of Gothenburg, Gothenburg, Sweden; 9 Hong Kong Center for Neurodegenerative Diseases, Hong Kong, China; 10 Centre for Age-Related Diseases, Stavanger University Hospital, Stavanger, Norway

**Keywords:** Parkinson's disease, Parkinson's disease

## Abstract

Early identification of cognitive impairment in Parkinson’s disease (PD) has important clinical and research implications. The aim of our study was to investigate the role of plasma tau phosphorylated at amino acid 181 (p-tau181) and plasma neurofilament light chain (NfL) as biomarkers of cognition in PD. Baseline concentrations of plasma p-tau181 and NfL were measured in a cohort of 136 patients with PD and 63 healthy controls (HC). Forty-seven PD patients were followed up for up to 2 years. Cross-sectional and longitudinal associations between baseline plasma biomarkers and cognitive progression were investigated using linear regression and linear mixed effects models. At baseline, plasma p-tau181 concentration was significantly higher in PD subjects compared with HC (*p* = 0.026). In PD patients, higher plasma NfL was associated with lower MMSE score at baseline, after adjusting for age, sex and education (*p* = 0.027). Baseline plasma NfL also predicted MMSE decline over time in the PD group (*p* = 0.020). No significant association between plasma p-tau181 concentration and baseline or longitudinal cognitive performance was found. While the role of p-tau181 as a diagnostic biomarker for PD and its relationship with cognition need further elucidation, plasma NfL may serve as a feasible, non-invasive biomarker of cognitive progression in PD.

## Introduction

Parkinson’s disease (PD) is a complex syndrome characterised by several motor and non-motor symptoms^1^. Recently, several subtypes and specific personalised therapeutic approaches based on specific non-motor symptoms profiles have been described, including a cognitive or cholinergic subtype, which is characterised by the presence of cognitive dysfunction^2,3^.

It is well established that cognitive impairment (CI) has a major impact on patient’s quality of life, functioning and health-related costs^4,5^.

The early identification of those individuals with PD who will develop CI therefore has pivotal clinical and research implications, and several studies have focused on the predictive value of clinical features as well as cerebrospinal fluid (CSF) and imaging biomarkers^4,6^. The identification of reliable, cheap and non-invasive biomarkers able to predict the cognitive progression in PD still represents a great unmet need^7^.

Neuropathologically, Parkinson’s disease dementia (PDD) is characterised by neuronal loss and the presence of diffuse α-synuclein pathology, especially in the limbic area and in the neocortex^8^. In addition, about 50% of PDD patients’ post-mortem brains showed Alzheimer’s disease (AD) like pathology, with extracellular β-amyloid plaques in cortical and subcortical regions, as well as intracellular hyperphosphorylated tau (p-tau) deposition in the hippocampal and neocortical regions in two-thirds of cases^9,10^. The measure of tau species in the CSF provides invaluable information for the diagnosis of AD, with tau phosphorylated at threonine 181 (p-tau181) being one of the most studied, due to its high specificity for tau pathology and correlation with amyloid β pathology^11^.

In accordance with post-mortem observations, low amyloid­β(1–42) (Aβ42) concentration in CSF has been associated with the development of mild cognitive impairment (MCI) or PDD^12^, while evidence for an association between CSF total tau or p­tau concentration and MCI or dementia has been limited mostly to cross­sectional studies^13^. Despite the encouraging results, especially for CSF Aβ42, the feasibility of collecting routine CSF biomarkers in the clinical setting for PD patients is limited.

Recently, new assays for the detection of p-tau species in plasma have been developed and validated, showing that plasma p-tau181 can discriminate AD patients from controls^14,15^ as well as AD patients from those with frontotemporal dementia (FTD)^16^. In addition, plasma p-tau181 concentration has been shown to be significantly higher in individuals with PD and atypical parkinsonian disorders (APD) compared to healthy controls^17^, and the biomarker has recently been shown to predict cognitive decline in patients with dementia with Lewy bodies (DLB)^18^. These findings are promising and suggest plasma p-tau181 has potential as a biomarker for cognitive decline in PD.

Neurofilament light chain (NfL) is a component of neurofilaments, a structural part of neuronal axons which determines axonal calibre^19^. NfL is a disease non-specific biomarker that reflects the level of neuronal and axonal damage, offering the opportunity to monitor disease progression and severity, irrespective of the underlying cause^20^. CSF NfL is a marker of neurodegeneration, and particularly high CSF NfL concentrations have been observed in amyotrophic lateral sclerosis, FTD and APD^21,22^. Additionally, CSF NfL correlates with annual Mini-Mental State Examination (MMSE) score decline, especially in AD and FTD^21^. In PD patients, CSF NfL has been shown to be higher in patients with worse motor function and cognitive impairment, even if the baseline concentration of NfL does not seem to predict conversion to dementia in cognitively intact PD patients^20^, and studies have demonstrated that high CSF NfL in early PD predicts subsequent conversion to PDD, especially when combined with low CSF Aβ42, and high CSF heart fatty acid-binding protein^23^.

Ultrasensitive Single molecule array (Simoa) methods to measure blood NfL concentration have been recently developed and used with success to discriminate PD from APD^22,24^. Moreover, higher plasma NfL concentration has been found in PDD patients compared with non-demented PD patients^25,26^. Some evidence has also been generated that suggests blood NfL concentration can predict cognitive decline in PD^26,27^. Therefore, further studies are needed to confirm the prognostic value of plasma NfL for CI in PD, and to compare it with p-tau181.

Given the potential of blood p-tau181 and NfL to predict cognitive decline in PD, the aim of this study is to investigate the association of plasma p-tau181 with cognitive performance in patients with PD, and to compare its utility as a prognostic marker for CI both alongside and relative to NfL.

## Results

### Baseline characteristics of the sample

The characteristics of the sample are shown in Table 1. The PD (*n* = 94) and healthy controls (HC) (*n* = 63) groups did not differ significantly in age or MMSE baseline scores, but the PD group included more men than the HC group (*P* = 0.008) and had more years of education (*P* = 0.022).

**Table 1:** Baseline characteristics of the cohort.

	HC	PD	*P* value
Total, *n* (%)	63 (40.1)	94 (59.9)	
Sex			
Female, *n* (%)	29 (46)	24 (25.5)	**0.008**
Male, *n* (%)	34 (54)	70 (74.5)	
Age	64.06 (7.61)	63.80 (11.24)	0.870
Years of education	14.4 (3.2)	15.8 (4.4)	**0.022**
Disease duration, years	–	6.07 (5.61)	–
Hohen&Yahr stage (1/2/3/4), *n*	–	14/48/30/2	–
MMSE	29.17 (1.09)	29.29 (1.20)	0.253
NMSS Total	–	42.97 (31.54)	–
NMSS Domain 5	–	3.87 (6.59)	–
Plasma p-tau181, pg/mL^a^	1.95 (1.58)	2.34 (1.34)	**0.026**^b^
Plasma NfL, pg/mL^c^	29.13 (16.26)	30.51 (22.55)	0.847^b^

Data are expressed in mean (standard deviation), where not otherwise specified. Bold values denote statistical significance at the *P* < 0.05 level.

*HC* healthy controls, *PD* Parkinson’s disease, *LEDD* Levodopa Equivalent Daily Dose, *MMSE* Mini-Mental State Examination, *NMSS* Non-Motor Symptoms Scale, *p-181* phosphorylated tau 181, *NfL* neurofilament light chain, *pg/mL* picograms per millilitre.

^a^Available for 62 HC and 90 PD.

^b^ANCOVA test including age and sex as covariates.

^c^Available for 57 HC and 90 PD.

### Group differences in plasma p-tau181 and NfL between PD and HC

**Fig. 1:**
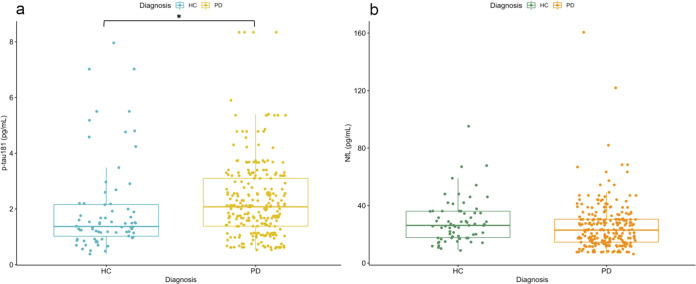
Plasma p-tau181 and NfL concentrations in the different diagnostic groups. Scattered boxplots showing plasma p-tau181 concentrations in HC and patients with PD (**a**) and NfL concentrations in the same groups (**b**). *Significant difference (*P* = 0.026), after adjusting for age and sex. PD Parkinson’s disease, HC healthy controls, pg/mL picograms per millilitre.

Plasma p-tau181 and NfL concentrations in the PD and HC groups are shown in Table 1 and Fig. 1. Patients with PD had higher baseline p-tau181 plasma concentration compared with HC (*P* value = 0.026, age and sex corrected) (Fig. 1a). When including the PD subgroup with limited available clinical data (*n* = 42), this difference remained statistically significant (*P* value < 0.001). There was no statistically significant baseline difference in plasma NfL concentration between HC and PD in both samples (Fig. 1b). Given the different size of the PD and HC groups, the difference in baseline p-tau181 and NfL were assessed with an additional Mann-Whitney test and the results remain unchanged: plasma p-tau181 concentrations were significantly higher in the PD group (*P* value = 0.002) and there was no statistically significant difference in plasma NfL concentrations between the two groups.

### Baseline association of plasma p-tau181 and NfL with MMSE scores and other clinical features

**Fig. 2:**
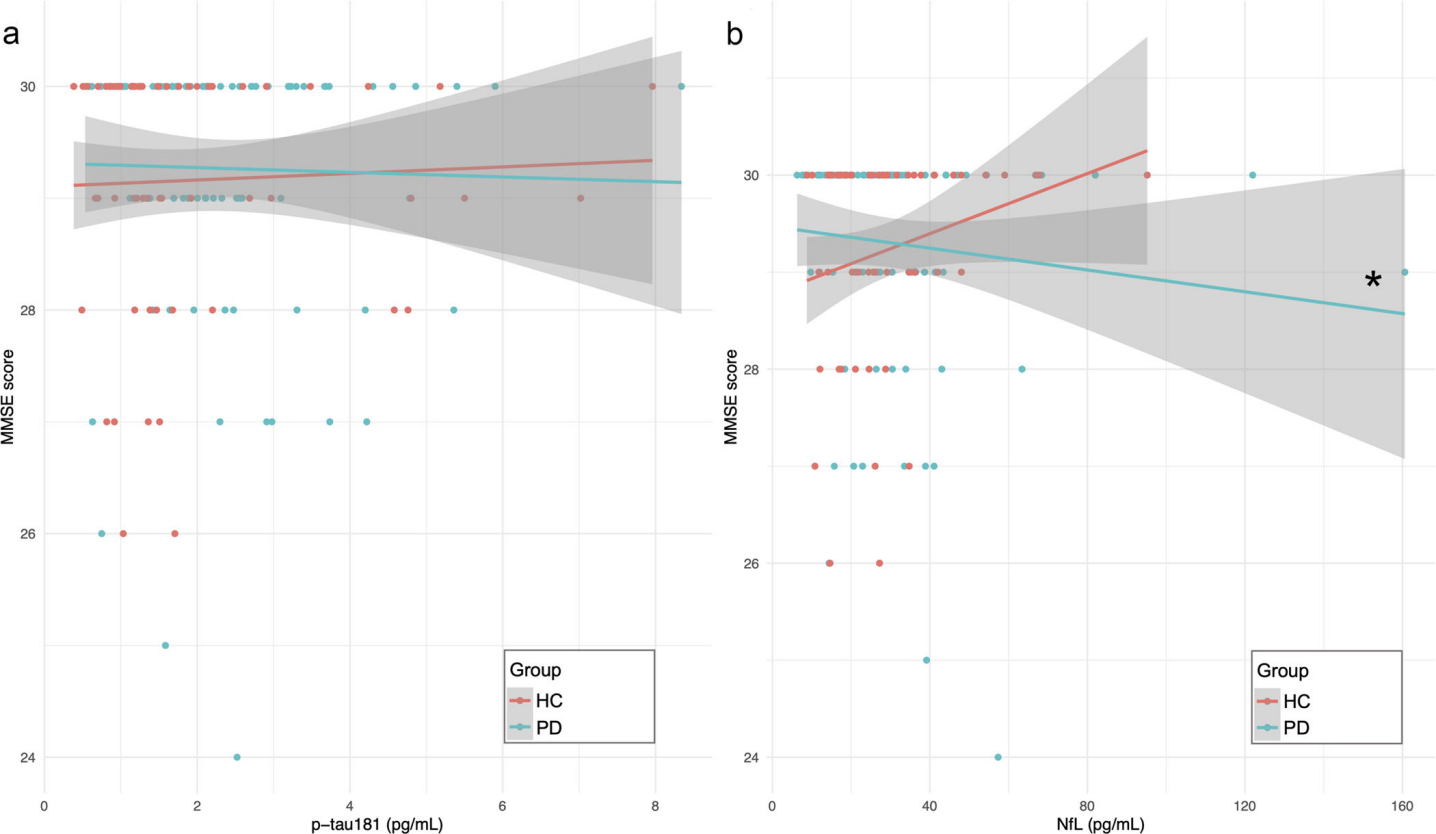
Association of baseline MMSE scores with plasma p-tau181 and NfL concentrations. Graphs representing MMSE score data points and regression lines in the PD group (blue line) and HC group (pink line) as function of plasma p-tau181 concentrations (**a**) and plasma NfL concentrations (**b**). *Significant linear regression model, with MMSE scores as dependent variables and plasma NfL as predictor, while adjusting for age, sex and years of education (Standardised *β* = 0.240, *P* = 0.027, overall regression model: R2 = 0.152, *P* = 0.002). MMSE Mini-Mental State Examination, PD Parkinson’s disease, HC healthy controls, pg/mL picograms per millilitre.

In the PD group with available clinical data (*n* = 94), higher baseline plasma NfL concentrations were associated with lower baseline MMSE scores (Standardised *β* = 0.240, *P* = 0.027, overall regression model: R2 = 0.152, *P* = 0.002) while adjusting for age, sex and years of education (Fig. 2a). No statistically significant association was found between baseline plasma p-tau181 and MMSE (Standardised *β* = 0.089, *P* = 0.413, overall regression model: R2 = 0.124, *P* = 0.003) (Fig. 2b).

In the HC group (*n* = 63), no statistically significant association was found between baseline plasma NfL and MMSE (Standardised *β* = −0.264, *P* = 0.099, overall regression model: R2 = 0.065, *P* = 0.553) (Fig. 2a) as well as between baseline plasma p-tau181 and MMSE (Standardised *β* = 0.046, *P* = 0.413, overall regression model: R2 = 0.019, *P* = 0.351) (Fig. 2b).

In the PD group, neither p-tau181 nor NfL concentrations at baseline were associated with the Non-motor Symptoms Scale (NMSS) Attention/Memory Domain score (Domain 5) (rho = −0.073, *p* = 0.500 for p-tau181, and rho = −0.083, *p* = 0.445 for NfL) (Supplementary Fig. 1a, d).

Baseline NfL concentration was associated with disease duration (rho = 0.261, *p* = 0.013) but not with Hoehn and Yahr (HY) stage (rho = 0.029, *p* = 0.784) (Supplementary Fig. 1e, f). No significant associations between baseline p-tau181 and disease duration (rho = 0.047, *p* = 0663) or HY stage (rho = −0.119, *p* = 0.264) were found (Supplementary Fig. 1b, c).

### Longitudinal association of baseline plasma p-tau181 and NfL with MMSE scores

**Fig. 3:**
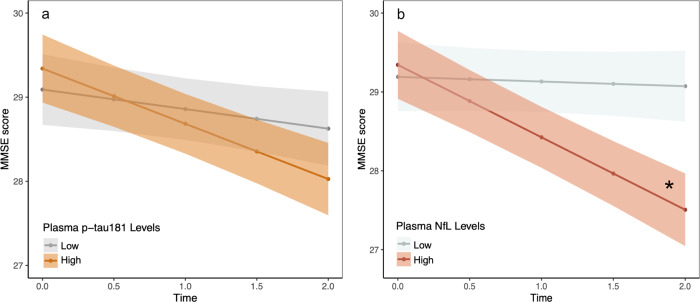
Longitudinal association of MMSE scores with baseline plasma p-tau181 and NfL concentrations. Graphs of estimated marginal models (solid lines) for the MMSE scores in relation to time in the PD group. The plots show different trajectories of MMSE over time (expressed in years from baseline) based on high and low 50th percentiles of plasma p-tau181 (**a**) and plasma NfL (**b**) concentrations, while correcting for age, sex, and years of education. The coloured ribbons represent the 95% confidence interval around the averages. *Significant linear mixed effects model, with MMSE scores as dependent variables and plasma NfL as predictor, while adjusting for age, sex, and years of education MMSE Mini-Mental State Examination.

Results from the Linear Mixed Effect Models (LMEM) are shown in Table 2 and Fig. 3. In the PD subgroup with longitudinal measurements of MMSE (*n* = 47, follow-up up to 2 years, mean follow-up 0.95 years, SD 0.81), there was a significant interaction between time and baseline NfL concentration, meaning that high baseline plasma NfL concentration was associated with MMSE decline over time (*P* = 0.020), while adjusting for age, sex, and education. In the model, each unit (pg/mL) of baseline plasma NfL predicted an annual decline of −0.036 MMSE points (Table 2, Fig. 3b). No significant association was found between baseline plasma p-tau181 concentration and longitudinal MMSE change (*P* = 0.376) (Table 2, Fig. 3a). Plots for LMEM models uncorrected for age, sex, and years of education as well plots for typical subjects at minimum, mean and maximum age can be found in Supplementary Figs. 2 and 3, respectively.

**Table 2:** Association between baseline plasma p-tau181 (*p-tau181 Model)* and NfL (*NfL Model*) and longitudinal MMSE scores.

Fixed Effects	MMSE	
	Estimate (SE)	*P* value
*p-tau181 Model*		
(Intercept)	28.731 (1.778)	<0.001
Time (years)	−0.226 (0.300)	0.453
p-tau181 (pg/mL)	−0.101 (0.225)	0.651
Age	−0.023 (0.024)	0.350
Sex	−0.499 (0.669)	0.459
Education	0.053 (0.059)	0.377
Time * p-tau181	−0.097 (0.109)	0.376
*NfL Model*		
(Intercept)	28.667 (1.778)	<0.001
Time (years)	0.320 (0.372)	0.393
NfL (pg/mL)	0.006 (0.029)	0.827
Age	−0.009 (0.025)	0.731
Sex	−0.455 (0.658)	0.494
Education	0.071 (0.060)	0.239
Time * NfL	−0.036 (0.015)	**0.020**

*p-tau181* phosphorylated tau 181, *NfL* neurofilament light chain, *MMSE* Mini Mental State Examination, *SE* standard error, *pg/mL* picogram per millilitre. Bold values denote statistical significance at the *P* < 0.05 level.

## Discussion

The present study demonstrated that, in a cohort of people with PD, baseline plasma NfL concentration was independently associated with cognitive performance at baseline and independently predicted its decline over a follow-up time of up to two years. In contrast, although plasma p-tau181 concentration was increased in PD, associations with cognitive performance were not observed, either cross-sectionally or longitudinally.

NfL is a well-recognised disease non-specific marker of neuroaxonal damage, and its role as marker of neurodegeneration has been established in AD^21,28–30^. In PD, however, there is not enough evidence to support its use as early diagnostic biomarker^22^ and, similarly to our findings, some studies have failed to demonstrate a difference in plasma NfL among PD and HC^31,32^, despite its utility in discriminating PD from APD^24,30^. Nevertheless, both CSF and plasma NfL concentrations have been associated with a more severe neurodegenerative process^21,24,31,33^, similarly to what is found in APD or in PD with additional cognitive impairment, which in turn reflects a more widespread Lewy body pathology^4^. Additionally, an inverse correlation between NfL concentration and cognitive score has been shown in several neurodegenerative disorders^20–22,31^. Previous findings have also shown that high plasma NfL concentration correlates with poor cognition in PD and that PDD patients have higher plasma NfL concentration compared with non-demented PD patients^25,34^. In addition, a direct correlation has been demonstrated between NfL and the risk of cognitive decline or dementia in PD progression^26,34^. Our results align with these findings, adding a quantification to these changes, demonstrating an annual decline of -0.036 MMSE points for each NfL unit change. This suggests plasma NfL has potential as a biomarker of disease progression in PD, while its utility as a diagnostic marker is limited.

Previous studies investigated differences in plasma p-tau181 concentration in different neurodegenerative disorders associated with cognitive decline, specifically in AD and FTD^17,35,36^. A recent study has demonstrated that plasma p-tau species might serve as useful marker for AD co-pathology in DLB/PDD, and that high plasma p-tau181 concentration is associated with a more rapid cognitive decline over time^18^. On the other hand, previous studies failed in demonstrating a significant association between plasma p-tau181 and cognitive performance in PD^17,34,37^. Similarly, in our PD cohort, no association of baseline plasma p-tau181 with baseline MMSE scores or longitudinal MMSE progression was observed, potentially suggesting that, unlike in patients with DLB or PDD, the AD-like pathology burden in non-demented PD subjects might not be severe enough to drive changes in cognitive performance. Of note, cognition was not impaired in our cohort, and a somewhat more impaired cohort, or a longer observation period, might be needed to detect this association. Further studies with larger sample sizes and longer follow-up periods are needed to explore the potential role of p-tau181 as a prognostic marker of cognitive decline in PD. On the other hand, p-tau181 might be better suited to assess cognitive progression in patients with more substantial AD-like pathology, such as patients with DLB or PDD.

In addition, our baseline findings of higher plasma p-tau181 concentration in the PD group compared with controls and non-significant differences in plasma NfL concentration might reflect what has been showed in a recent study assessing the temporal trajectories of fluid biomarkers in a de novo PD cohort: at baseline, CSF p-tau but not serum NfL concentration was significantly higher in patients than in the control group while, over time, the increase observed in NfL concentration was much higher than the increase observed in p-tau concentration^38^. The mechanisms behind the different temporal trajectories of these two biomarkers are, however, still unclear and further studies focusing on the neuropathological processes associated with changes in fluid biomarkers are needed.

Our study has several limitations. First, the small sample size of our cohorts, including the limited number of subjects with a longitudinal follow up, might prevent our results from generalisation, thus larger studies are needed to confirm our findings. Second, although the longitudinal nature of this study is a strength, the attrition rate and the length of follow-up may have underestimated the extent and severity of cognitive decline that we observed over time and, subsequently, could have limited the possibility for us to detect potential cognitive changes associated with p-tau181. Third, cognitive status was assessed using MMSE, which is a global measure of cognition that does not provide detailed information on the individual cognitive domains. However, we have previously shown that MMSE is a sensitive marker of cognitive change in PD^39^. Nevertheless, despite our cohort of PD patients was not overtly cognitively impaired according to the available MMSE scores, we acknowledge that subtle changes in specific cognitive domains in these subjects might have not been detected due to lack of a detailed neuropsychological testing. Therefore, more domain-specific associations between plasma p-tau181 and NfL might have been undetected and only future studies using extensive cognitive tests will be able to investigate correlations of these biomarkers with specific cognitive aspects of PD. Finally, patients’ diagnosis was based on clinical judgement, and none of the participants had a biomarker or post-mortem pathological confirmation. However, diagnoses were made by movement disorders experts and confirmed at follow-up for a sub-group of patients, a procedure which has been shown to have a high diagnostic accuracy for PD^40^.

Despite these limitations, our results are encouraging and pave the way for the use of plasma NfL as non-invasive and feasible predictive biomarker of cognitive impairment in PD. Further studies with larger sample size and longer longitudinal follow-up are needed in order to clarify the role of plasma p-tau181 in PD, and to establish and validate plasma NfL cut-offs that can be used in clinical practice.

## Methods

### Sample cohorts

Data used in the preparation of this article were obtained from a cohort of patients with a diagnosis of probable idiopathic PD according to the UK Brain Bank criteria^41^ from the Parkinson’s Foundation Centre of Excellence at King’s College Hospital in London, UK. PD patients were enroled in the Non-motor International Longitudinal Study (NILS). The NILS (https://www.gsttbrc.com/NILS) is a prospective cohort study designed to assess the range, nature, and natural history of non-motor symptoms in PD over time and has been adopted as a national study by the National Institute of Health Research in the United Kingdom. Patients are assessed at baseline and annually after inclusion. Exclusion criteria included age of PD onset <21 and insufficient archived plasma for analysis. Additional samples from 42 PD patients recruited at the same centre (Parkinson’s Foundation Centre of Excellence at King’s College Hospital in London, UK) were retrieved from archives in the South London and Maudsley Biomedical Research Centre (BRC), together with plasma samples from HC for comparison analyses. Clinical data for the additional PD subjects were limited and, as such, the samples were used only in a secondary analysis.

The NILS study was authorised by local ethics committees (NRES South East London REC, 10084, 10/H0808/141). Plasma samples from consented HC were retrieved from archives in the South London and Maudsley Biomedical Research Centre (BRC) (NRES South Central - Oxford C Bristol REC, 15/SC/0388). All subjects gave written consent prior to study procedures in accordance with the Declaration of Helsinki and all patient data were anonymised and coded.

### Clinical data

Data extracted from 94 PD patients and 63 HC included demographics, such as age, sex, years of education and, for PD subjects, disease duration and HY stage^42^. HY classification reflects the global severity of the disorder and represents the historically most relevant instrument for this purpose. It is a useful tool for classifying patients with PD according to a combination of motor symptoms and disability. For these reasons, the HY could be considered a key element for motor grading of PD and is now considered a key quality standard for clinical assessment of PD patients^43,44^.

Cognitive function was assessed using the MMSE^45^, as this was the only cognitive screening measure available in our cohort. This tool, however, has been shown to be a good measure of cognitive decline in PD^39^. Non-motor symptoms were assessed using the NMSS, a tool designed to comprehensively assess a wide array of non-motor manifestations associated to PD across all stages of disease severity^46^. The scale is a rater-based tool and is composed of 30 items grouped in nine domains (cardiovascular, sleep/fatigue, mood/apathy, perceptual problems/hallucinations, attention/memory, gastrointestinal, urinary, sexual function, miscellaneous). International validation studies of the NMSS have been carried out^46,47^ and has also been utilised for measuring the effect of NMS burden on patients’ quality of life^48^.

A subgroup of subjects with PD (*n* = 47) had annual longitudinal cognitive assessments for up to 2 years.

### Plasma p-tau181 and NfL measurements

Blood samples were collected in EDTA tubes and then centrifuged at 3500 revolutions per minute for 10 min. The obtained plasma (supernatant) was then aliquoted and frozen at –80 °C before measurements. All samples were visually inspected for haemolysis. Plasma p-tau181 concentration was measured at King’s College London using the commercially available Simoa® pTau-181 V2 Advantage Kit (Quanterix; 103714). Plasma was diluted 1:4 and read on the HD-X analyser (Quanterix). Data acquisition spanned 5 analytical runs, the lower limit of quantification (LLOQ) for this assay was 0.127 pg/mL and the coefficient variation for inter and intra-assay variability was 7.51% and 7.69% respectively. Plasma NfL concentration was measured using the commercially available NF-Light kit on an HD-X analyser at the UK DRI Fluid Biomarker Laboratory, London, UK in one round of experiments using one batch of reagents. Intra-assay coefficients of variation were <5%. The limit of detection was 0.038 pg/mL and LLOQ was 0.174 pg/mL. To reduce the risk of any potential bias, the analysts conducting the assays were blinded to patient status from each sample.

### Statistical analysis

Baseline characteristics of the cohorts were reported using proportions for categorical variables and means and standard deviations for continuous variables. Normality distribution assumption was tested with Shapiro-Wilk tests. To achieve normal distribution, plasma p-tau181 and NfL concentrations were log10-transformed and MMSE score was transformed using the squared root of 30 minus the MMSE score. Group comparisons (PD vs HC) for quantitative variables were performed using independent *t* tests, Mann-Whitney U-tests or ANCOVAs, distribution dependent. Chi-squared test were used to assess group differences in categorical variables.

The cross-sectional correlations of plasma p-tau181 and NfL concentrations with cognition (MMSE) in PD subjects and HC were tested with linear regression models, including MMSE as dependent variable and either p-tau181 or NfL, together with age, sex and years of education as predictors. Within PD patients, correlations between baseline p-tau181 and NfL with disease duration, HY stage and the NMSS Attention/Memory Domain (Domain 5) scores were assessed using Spearman’s rank correlation. Due to their suitability to repeated-measures analyses and their ability to handle missing data using maximum likelihood estimation, LMEM were used to test whether baseline p-tau181 and NfL concentrations predicted longitudinal cognitive decline in a subgroup of subject with PD who had available longitudinal data. These analyses included longitudinal MMSE scores as dependent variables in addition to p-tau181 or NfL, time, age, sex, and years of education as fixed effects. The models included all main effects, the interaction between p-tau species and time, and random effects for intercepts.

Statistical analyses were performed using the Statistical Package for the Social Sciences, version 27.0 (IBM Corp., Armonk, NY, USA) and R software, version 3.6.1 (R Foundation for Statistical Computing, Vienna, Austria, r-project.org,). Significance threshold was set to *p* < 0.05, where applicable values are given for two-tailed tests.

## Supplementary information

Supplementary Figures

## Data Availability

All data and materials that support the findings of this study are available from the corresponding author upon reasonable request.
