# Supplementary material for: Plasma p-tau181, neurofilament light chain and association with cognition in Parkinson’s disease
Source: NPJ Parkinsons Dis. 2022 Nov 12;8:154. doi: 10.1038/s41531-022-00384-x (PMC9653432; doi:10.1038/s41531-022-00384-x)
Supplement: Supplementary file 1 — Supplementary Figures [file 41531_2022_384_MOESM1_ESM.docx]

**Plasma p-tau181, neurofilament light chain and association with cognition in Parkinson’s disease**

**Supplementary Figures**


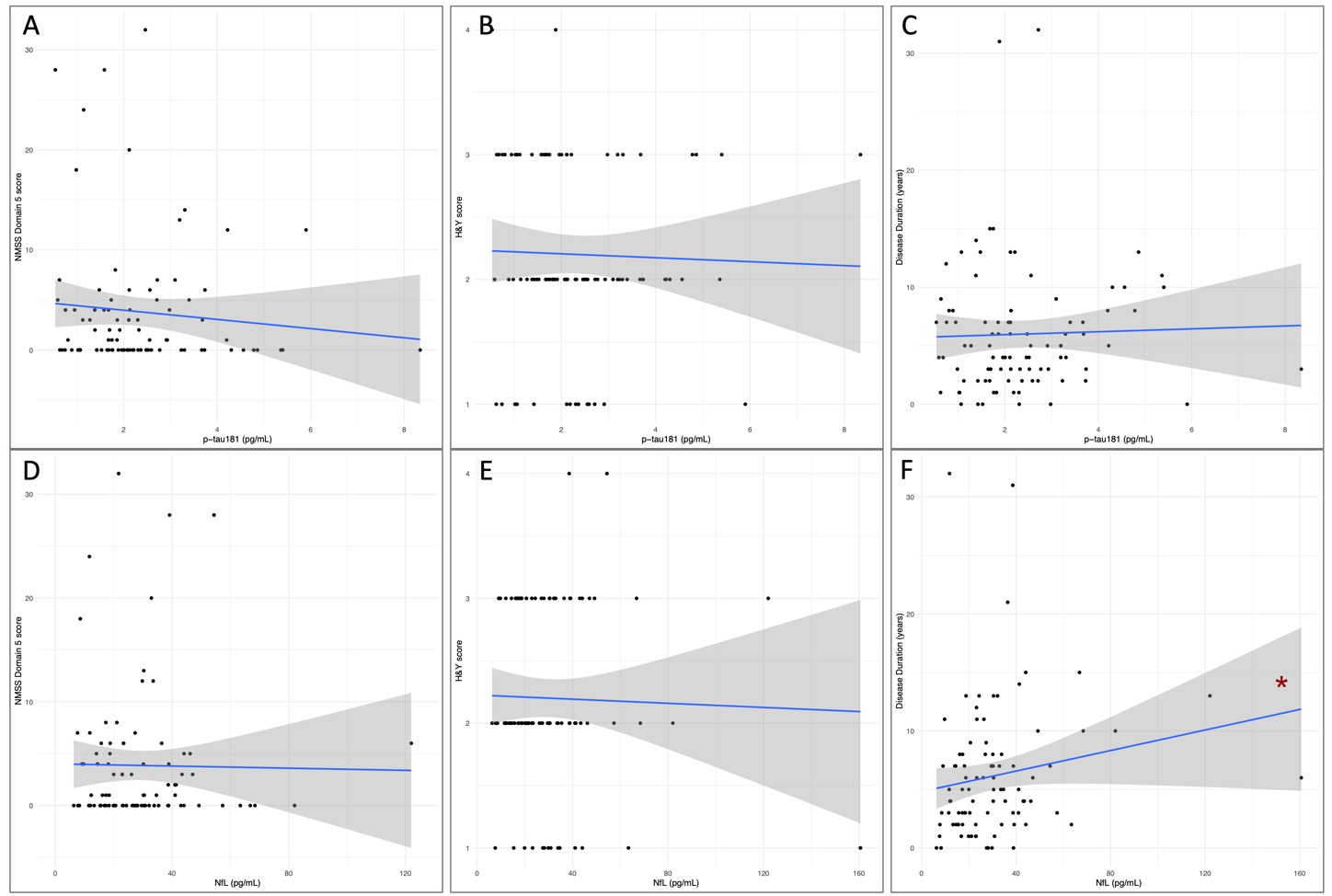


**Supplementary Figure 1.** Graphs representing NMSS Domain 5 (Attention/Memory) scores (**panel A, D**), H&Y scores (**panel B, E**) and disease duration (**panel C, F**) with regression lines in the PD group as function of plasma p-tau181 concentrations (**panel A-C**) and plasma NfL concentrations (**panel D-F**). The grey area represents the 95% confidence interval around the averages.
*Significant correlation between disease duration and plasma NfL concentrations (Spearman’s rho = 0.261, p = 0.013).
Abbreviations: pg/mL: picograms per millilitre; NMSS: Non-motor Symptoms Scale; H&Y: Hoehn and Yahr scale.


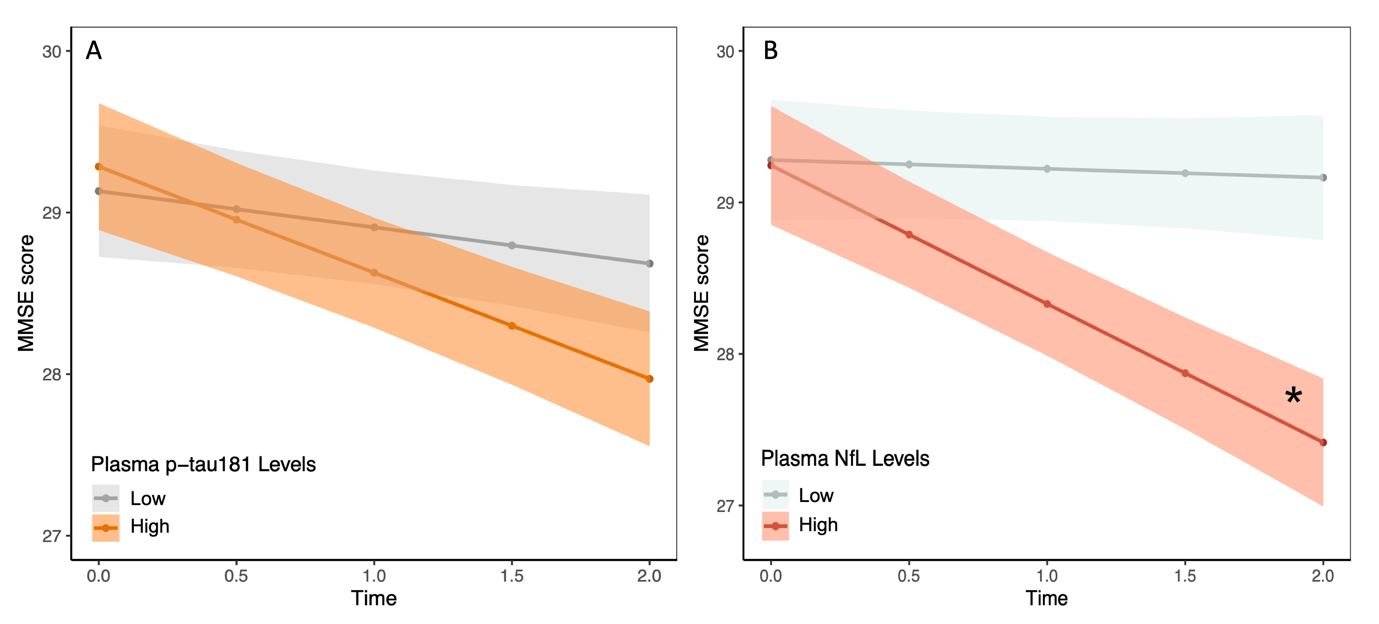


**Supplementary Figure 2.** Graphs of estimated marginal models (solid lines) for the MMSE scores in relation to time in the PD group. The plots show different trajectories of MMSE over time (expressed in years from baseline) based on high and low 50th percentiles of plasma p-tau181 (panel A) and plasma NfL (panel B) concentrations, with no correction for age, sex and years of education. The coloured ribbons represent the 95% confidence interval around the averages.
*Significant linear mixed effects model, with MMSE scores as dependent variables and plasma NfL as predictor, while adjusting for age, sex and years of education.
Abbreviations: MMSE, Mini-Mental State Examination.

**
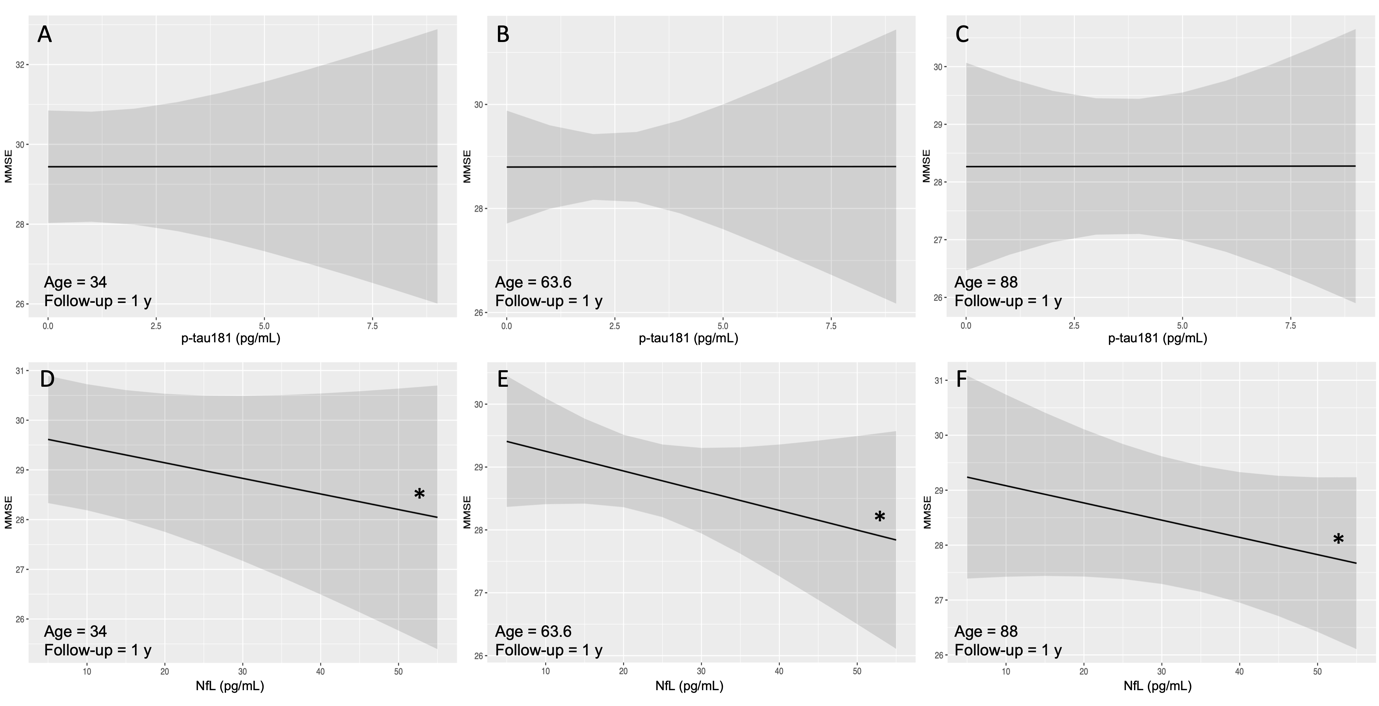
**

**Supplementary Figure 3.** Graphs of estimated marginal models (solid lines) for the MMSE scores in relation to plasma concentrations of p-tau181 (panels A-C) and NfL (panels D-F) for three typical subjects at 1 year of follow-up: **panel A, D)** subject at the youngest age recorded in our sample (34); **panel B, E)** subject at the mean age of 63.6 and **panel C, F)** subject at the oldest age recorded in our sample (88). The grey area represents the 95% confidence interval around the averages.
*Significant linear mixed effects model, with MMSE scores as dependent variables and plasma NfL as predictor, while adjusting for age, sex and years of education.
Abbreviations: MMSE, Mini-Mental State Examination; pg/mL, picograms per millilitre.
